# A137 BALLOON-ASSISTED ENDOSCOPY FOR SMALL BOWEL BLEEDING – A REVIEW

**DOI:** 10.1093/jcag/gwad061.137

**Published:** 2024-02-14

**Authors:** G Malik, J Y Guo, B Halloran, S Zepeda-Gomez

**Affiliations:** University of Calgary Cumming School of Medicine, Calgary, AB, Canada; University of Alberta, Edmonton, AB, Canada; University of Alberta, Edmonton, AB, Canada; University of Alberta, Edmonton, AB, Canada

## Abstract

**Background:**

Gastrointestinal (GI) bleeding is a potentially life-threatening medical emergency requiring immediate medical attention. Obscure GI bleeding (OGIB) Is defined as GI bleeding with no obvious culprit lesion found on initial upper or lower endoscopy. Further investigations are needed as 75% of those cases originate in the small bowel. The advent of small bowel endoscopy, consisting of video capsule endoscopy (VCE) and device-assisted enteroscopy (DAE) has allowed clinicians to endoscopically visualize the entire GI tract and perform therapeutic interventions. These devices include balloon assisted endoscopy (BAE) and Spiral Endoscopy (SPE).

**Aims:**

The objectives of this review are to analyze the definitions, etiologies, and risk factors of OGIB. Furthermore, we reviewed the diagnostic and therapeutic yields of DAE and its safety profile. Due to the adverse effects and recall of SPE, we only report here on BAE.

**Methods:**

A literature review was conducted using PubMed and OvidSP (Medline) to identify articles on the use of VCE and DAE for OGIB. Keywords included a combination of gastrointestinal bleeding with occult, obscure, active and inactive, small bowel bleed, diagnostic yield, therapeutic yield, and rebleeding rate. The information was obtained via systematic review and meta-analysis, retrospective studies, and case reports.

**Results:**

A total of 32 papers were reviewed. The most common causes of OGIB reported were vascular lesions (23-47%), inflammatory lesions (12-16%), and tumors (5-16%). The risk factors associated with OGIB include age, cardiac disease, liver cirrhosis, chronic kidney disease, and hereditary vascular diseases. The overall diagnostic and therapeutic yields of BAE in OGIB were 76.3% and 49.5%, respectively. Factors that increased the yield included performing an initial VCE (increase by 7%) and performing the procedure within the first 10 days after the initial bleeding episode (87.5%) as compared to after 10 days (11.1%). Recurrent bleeding can occur in up to 46% of patients. Risk factors for rebleeding include cirrhosis, overt bleeding symptoms, and presence of multiple vascular lesions. Associated adverse effects in DAE were minor in 9.1% of patients and severe in less than 2%. These include bleeding (0.8%), perforation (0.3%), and pancreatitis (0.3%).

**Conclusions:**

OGIB is a challenging clinical entity. BAE now allows clinicians to diagnose and treat culprit lesions in the small bowel with reasonable success and a strong safety profile, however timing and prior VCE can further optimize this. Clinical decisions and follow up should be made based around existing risk stratification tools and scores. Further prospective research is needed to determine optimal timing of DAE from presentation of OGIB that would increase diagnostic and therapeutic yields.

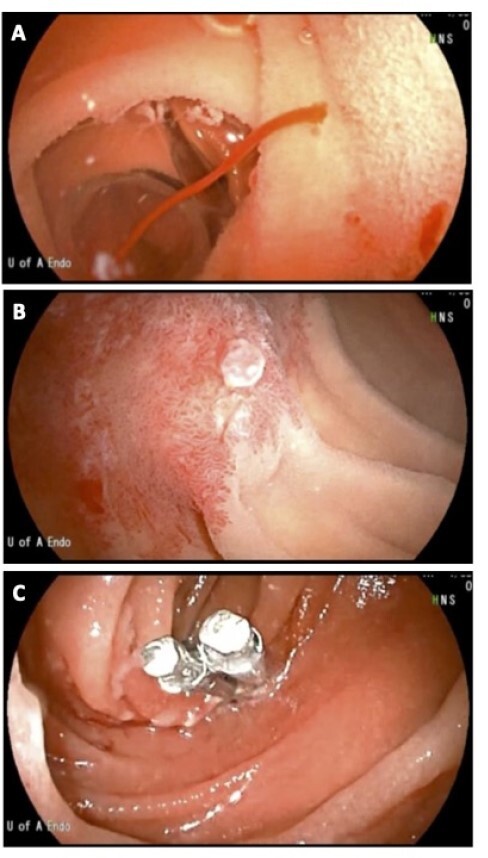

Figure 1. A and B) Endoscopic images of small bowel vascular lesion. C) Bleeding vascular lesion treated with hemostatic clips.

**Funding Agencies:**

None

